# Human Umbilical Cord Blood Derived-Mesenchymal Stem Cells Alleviate Dextran Sulfate Sodium-Induced Colitis by Increasing Regulatory T Cells in Mice

**DOI:** 10.3389/fcell.2020.604021

**Published:** 2020-11-24

**Authors:** Ying Li, Ke Ma, Luping Zhang, Hong Xu, Nan Zhang

**Affiliations:** ^1^Department of Gastroenterology, The First Hospital of Jilin University, Changchun, China; ^2^Department of Pediatrics, The First Hospital of Jilin University, Changchun, China

**Keywords:** human umbilical cord blood, mesenchymal stem cells, regulatory T cells, colitis, dextran sulfate sodium

## Abstract

Inflammatory bowel disease (IBD), which main clinical manifestations include abdominal pain and diarrhea occurring repeatedly, is a kind of autoimmune disease. It has been reported in preceding studies that mesenchymal stem cells (MSCs) can reduce inflammation by regulating the function of immune cells. But studies about the interaction between MSCs and adaptive immune cells, especially in IBD models, are insufficient. Therefore, the objective of this research was to estimate the therapeutic effects of MSCs from human umbilical cord blood (hUCB-MSCs) in an IBD model of rodent and to clarify the therapeutic mechanisms of hUCB-MSCs. Dextran sulfate sodium (DSS) was used to induce colitis in rodent. Mice with colitis were treated with intraperitoneal infusions of hUCB-MSCs and evaluated for mortality and diverse disease symptoms containing weight reduction, diarrhea, and bloody stools. The levels of histopathologic severity and generation of regulatory T cells (Treg) were also determined. Treatment with hUCB-MSCs ameliorated the clinical and histopathologic severity of acute and chronic colitis in mice. Furthermore, T cell infiltration into the inflamed colon was significantly decreased (*p* = 0.0175), and Foxp3^+^ cells were substantially higher in the hUCB-MSC group than that of the DSS group. Our results suggest that hUCB-MSCs are able to alleviate inflammation via adding Foxp3^+^ Tregs in an IBD model of mouse. As a result, these findings suggest the opportunity of hUCB-MSC being applied to patients with IBD.

## Introduction

Inflammatory bowel disease (IBD) is a kind of recurrent-and-remittent disease, which consists of ulcerative colitis and Crohn’s disease, and characterized by intestinal mucosal destruction. Its pathogenesis is related to dysfunctional immune cells and changed inflammatory state. This immune irregularity and inflammation causes mucosal destruction of colon in the end, and some patients even had distal small bowel involvement (Anderson et al., 2013). An imbalance between T effectors and T regulators (Tregs) causes the expansion of self-reactive T cells and subsequent inflammation (Bouma and Strober, 2003). Existing treatments for IBD mainly consist of corticosteroids, immunosuppressants and biological agents, but it is a great pity that they are non-specific and do not work for all patients, furthermore, they have many side effects. Ultimately, surgical removal of the colon is the only option (Gonzalez et al., 2009; Zhu et al., 2018); thus, there is a clear need for novel therapeutic approaches. It has been reported that human mesenchymal stem cells (MSCs) can promote epithelial regeneration, facilitate angiogenesis, and reduce inflammation, and has also been reported that they have wide bioengineering applications (Chang et al., 2018; Wang et al., 2018; Yang et al., 2020). Therefore, using MSCs presents a unique cell-based strategy for the treatment of IBD with the potential to maintain intestinal homeostasis (Qiu et al., 2017).

An increasing number of evidences has proved that MSCs from bone marrow (BMSCs) display profound immunomodulatory and anti-inflammatory capabilities (Selmani et al., 2008). The immunosuppressive ability of BMSCs is major histocompatibility complex non-restrictive, which accounts for the supposed ‘immune-privileged’ state of MSCs that enables them to be successfully carried out in xenogenetic transplants in some animal models (Chamberlain et al., 2007). BMSCs manifest immunosuppressive abilities mainly through suppressing proliferation and affecting function of immune cells, for instance, T and B lymphocytes, NK cells, and dendritic cells, furthermore, enhancing the amplification of CD25^+^Foxp3^+^ Tregs (Selmani et al., 2008; Zhang et al., 2009). Apart from bone marrow, other origins of human MSCs include adipose, umbilical cord blood, the gingiva, and the epidermis (Nauta and Fibbe, 2007; Zhang et al., 2009; Yang et al., 2019). Due to the difficulty in obtaining sufficient autologous BMSCs, human MSCs obtained from umbilical cord blood, which exhibit multidirectional differentiation, shorter proliferation time, low immunogenicity, easy extraction, and long survival time after transplantation have become a promising option for cell therapy (Zhang L. et al., 2018). Here, we characterize the curative effects of hUCB-MSCs in IBD models of mice and their ability to suppress the inflammatory reaction and restore immune tolerance *in vivo*.

## Materials and Methods

### Cell Preparation and Culture

Human umbilical cord blood derived-mesenchymal stem cells were gifted by Associate Prof. Chang Pengyu of First Bethune Hospital of Jilin University, Changchun, China. hUCB-MSCs were thawed and passaged upon reaching 80% confluence. All cultures were cultivated in an incubator at 37°C with 5% CO_2_ and atmospheric O_2_.

### Lymphocyte Proliferation Assay

Peripheral blood mononuclear cells (PBMCs) were obtained from health control by Ficoll density gradient centrifugation at 931 g for 30 min. For proliferation analysis, PBMCs were stimulated with 5 μg/mL phytohemagglutinin (PHA; Thermo Fisher Scientific, Shanghai, China, 00-4977-93) in 96-well plates (1 × 10^5^ PBMCs per well) for 4 days with or without hUCB-MSCs. The stimulation ratios were as follows: hUCB-MSC: PBMC = 1:1, 0.5:1, and 0.25:1. After 4 days, the proliferation of PBMCs was measured with CCK-8 kit (Beyotime, Shanghai, China, C0043).

CD4 and CD8 cells were sorted using Dynabeads FlowComp Human CD4 Kit (Thermo Fisher Scientific, 11361D) and Dynabeads FlowComp Human CD8 Kit (Thermo Fisher Scientific, 11362D), respectively, both derived from human PBMCs. 1 × 10^5^ carboxyfluorescein succinimidyl ester (CFSE) (Abcam, Shanghai, China, ab113853)-labeled CD4 or CD8 cells were stimulated in a 96-well plate with 10 μg/mL plate-bound CD3 antibodies (BD, Shanghai, China, and 550368) plus 1 mg/mL CD28 antibodies (BD, 555726) for 4 days. At the beginning of the stimulation period, add half number of hUCB-MSCs to the relevant wells. CFSE dilution was used to observe proliferation of CD4 or CD8 cells via flow cytometry.

### Cell Culture With Interferon (IFN)-γ Treatment

Human umbilical cord blood derived-mesenchymal stem cells were seeded with 1 × 10^5^ cells per well in a 12-well plate. After overnight adherence, IFN-γ (at 0 and 20 ng/mL) (Absin Bioscience, Shanghai, China, abs00917) was added to the medium for 2 days, and then cells and cell culture supernatants were collected for qPCR and cytokine enzyme-linked immunosorbent assay (ELISA).

### Real-Time Quantitative PCR (RT-qPCR)

For reverse transcription, RNA was extracted using TRIzol Reagent (Invitrogen, United States, 15596018). RNA (1 μg) was reverse transcripted using HiScript II Q RT SuperMix (Vazyme, Nanjing, China, R223-01). qPCR was performed and analyzed using the Real-Time PCR Instrument [Applied Biosystems (ABI) 7300 PULAS, Thermo Fisher Scientific, United States] with ChamQ Universal SYBR qPCR Master Mix (Vazyme, Q711-02). Glyceraldehyde-3-phosphate dehydrogenase (GAPDH) was used for internal normalization. Primers used for qPCR were as follows: 5′-GACCACAGTCCATGCCAT CAC-3′ (forward) and 5′-TCCACCACCCTGTTGCTG TAG-3′ (reverse) for GAPDH; 5′-GCCCTTCAAGTGTTTCAC CAA-3′ (forward) and 5′-CCAGCCAGACAAATATATGCGA-3′ (reverse) for indoleamine-2,3-dioxygenase (IDO); 5′-TCAAGAT GTACGTGGTGGCC-3′ (forward) and 5′-CAGAAAGGA GTAGACGAAGCC-3′ (reverse) for prostaglandin E_2_ (PGE2); 5′-CCCAGGGACCTCTCTCTAATC-3′ (forward) and 5′-GCT ACAGGCTTGTCACTCGG-3′ (reverse) for tumor necrosis factor (TNF)-α; 5′-GGACACCAACTATTGCTTCAGCTCC-3′ (forward) and 5′-AGGCTCCAAATGTAGGGGCAGG-3′ (reverse) for transforming growth factor (TGF)-β1.

Cell culture supernatants of hUCB-MSCs were collected after 2 days with IFN-γ (0 and 20 ng/mL) stimulation. Three cytokines, including IDO, PGE2, and TGF-β1 were detected using the Human IDO ELISA kit (Biorbyt, United Kingdom, orb563242), Human PGE2 ELISA Kit (Biorbyt, orb564775), and Human TGF-β1 ELISA kit (ScienCell, United States, EK0513) following the supplier’s recommendations.

### Induction and Treatment of Colitis

All mice were maintained under specific pathogenic-free conditions as follows: room temperature (20 to 24°C), humidity (35 – 55%), light/dark cycle (12/12 h), unlimited food and water. The Animal Ethics Committee of the First Hospital of Jilin University (Changchun, China) approved all procedures using mice in this study.

Adding 3% dextran sulfate sodium (DSS, MP Biomedicals, United States, 02160110-CF) to the drinking water from days 1 to 8 contributed to induce acute colitis in C57Bl/6 mice (8-week-old, female, 18–22 g, Charles River, Beijing, China). On days 2 and 4, hUCB-MSCs (2 × 10^6^ per mouse) were injected intraperitoneally (IP) in the DSS + hUCB-MSC group. 200 μL PBS was injected IP into DSS group. Adding 3% DSS to the drinking water in two cycles induced chronic colitis in C57Bl/6 mice (Anderson et al., 2013), each cycle included 7 days with DSS, and subsequent 5 days with normal water. On days 2 and 4 of each cycle, mice in the DSS + hUCB-MSC group were injected with hUCB-MSCs IP (2 × 10^6^ cells per mouse). 200 μL PBS were injected IP into mice of DSS group. The severity of colitis was assessed daily by scoring the disease activity index (DAI), including weight loss, stool consistency, and the degree of intestinal bleeding (Wirtz et al., 2017). Acute colitis mice and chronic colitis mice were euthanized on days 10 and 25, respectively. Colon length was used as an indirect inflammatory symbol. Colon segments were processed for histopathological analysis or frozen in liquid nitrogen, and myeloperoxidase (MPO) activity was measured using MPO assay kit (Jiancheng Bioengineering Institute, Nanjing, China, A044). At the same time, spleen and mesenteric lymph nodes (MLNs) were collected for flow cytometry.

### Histology and Immunohistochemistry

Once euthanized, the same part of colon in all mice were immediately fixed, embedded, and sliced, and then hematoxylin and eosin staining were used to analyze the degree of inflammation. Inflammatory cell infiltration into the lamina propria and tissue damage were both graded blindly (Wirtz et al., 2017).

Paraffin was removed from colon sections, and then deparaffinized sections were hatched with anti-Foxp3 (Abclonal, Wuhan, China, A12051) and anti-CD4 (Abclonal, A0363) primary antibodies. Next, sections were incubated with a secondary antibody using the UltraSensitive SP (Rabbit) IHC Kit (Absin Bioscience, abs957) following the manufacturer’s instructions. Finally, slides were counterstained with hematoxylin and covered with neutral gum.

### Flow Cytometry

Human umbilical cord blood derived-mesenchymal stem cells were harvested and stained with fluorescein-conjugated antibodies for 30 min at 4°C and avoid light. After washed twice, cells were analyzed using BD FACSVerse Flow Cytometer (BD Biosciences, San Jose, United States) and FlowJo_V10. Antibodies used were as follows: human CD29 PE/Cy5 (BioLegend, CA, United States, 303005), human CD73 PE/Cy7 (BioLegend, 344009), human CD90 AF700 (BioLegend, 328120), human CD105 BV421 (BioLegend, 323219), human CD11b APC/Cy7 (BioLegend, 301341), human CD19 APC (BD Biosciences, 555415), human CD34 PE (BD Biosciences, 560941), and human CD45 APC/Cy7 (BD Biosciences, 557833).

Isolated lymphocytes (5 × 10^5^ cells) from the spleen and MLNs were analyzed using flow cytometry to evaluate the characterization of Tregs. Tregs were characterized with the following conjugated anti-mouse antibodies: CD4 Percp/Cy5.5 (BioLegend,100434), CD25 APC (BD Biosciences, 558643), and Foxp3 PE (BD Biosciences, 560408). Cells were fixed and permeabilized using fixation/permeabilization buffer kit (R&D Systems, FC009)for Foxp3 staining complying with the manufacturer’s instructions. The numbers of Treg gate represented the percentage of Treg in the CD4 gate in MLNs and spleens.

### Statistical Analysis

All data were expressed as the mean ± SD. For cell culture experiments, the non-parametric Mann–Whitney test was applied. To compare weight and DAI changes, the Wilcoxon matched-pairs signed-ranks test was applied. For survival rate, the Kaplan-Meier log-rank test was applied. In order to compare the statistical significance between groups, we used Tukey’s multiple comparison ANOVA test. All statistical analyses were performed using GraphPad Prism 7.0 statistical software. *p* < 0.05 was considered statistically significant.

## Results

### Characterization of hUCB-MSCs

**FIGURE 1:**
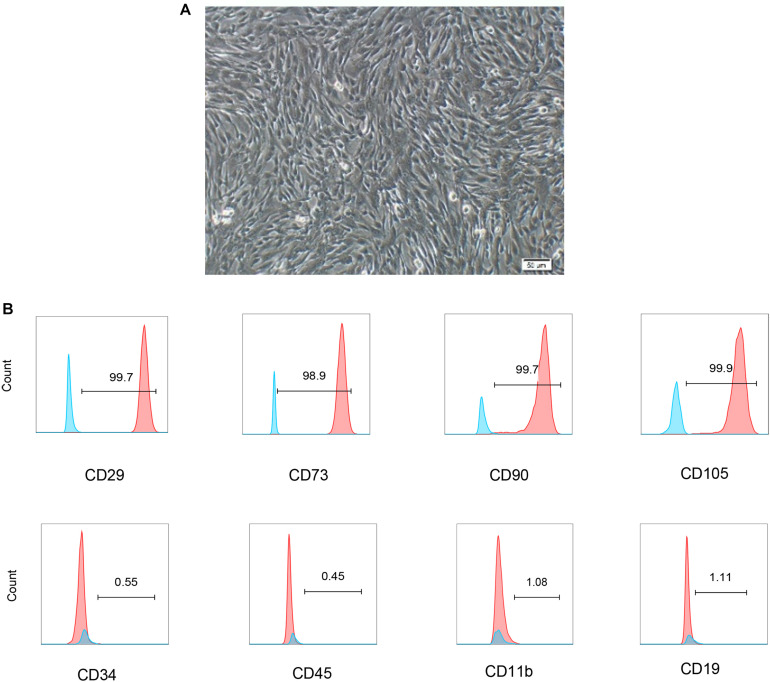
Characterization of hUCB-MSCs. **(A)** Representative micrograph showing the typical cell morphology of hUCB-MSC colonies of proliferating fibroblast-like mesenchymal cells. **(B)** The phenotype of hUCB-MSCs and the percentage of cells with positive cell surface markers (blue line was negative staining control, red line was specific staining of indicated antibody).

Human umbilical cord blood derived-mesenchymal stem cells displayed a fibroblast-like morphology (Figure 1A). The phenotype of hUCB-MSCs was detected using flow cytometry. Positive expression in CD29, CD73, CD90, and CD105, were found in cells. In addition, cells were negative for CD34, CD45, CD19, and CD11b (Figure 1B).

### hUCB-MSCs Inhibit T Lymphocyte Proliferation *in vitro*

**FIGURE 2:**
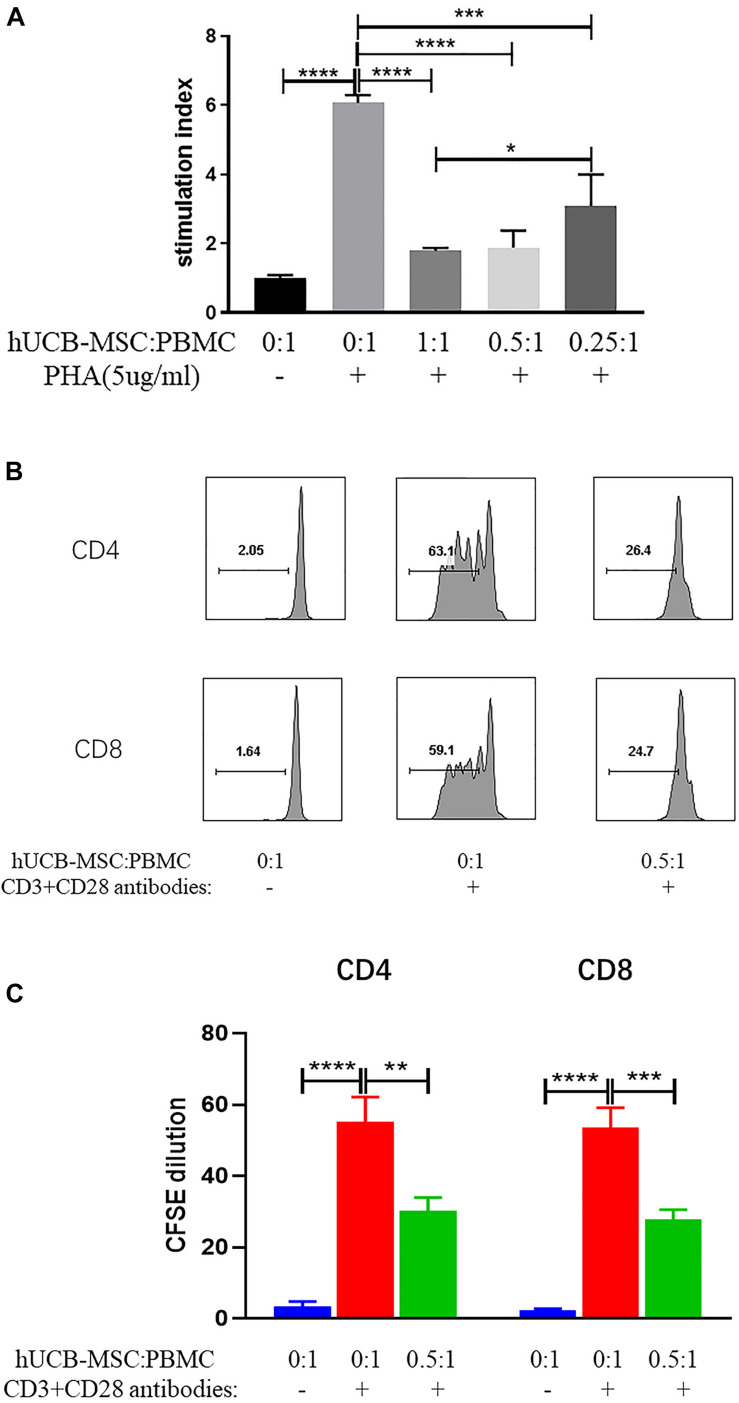
hUCB-MSCs are capable of suppressing T cell proliferation *in vitro*. **(A)** Inhibitory effects of hUCB-MSCs on PHA-stimulated PBMC proliferation. In the presence or absence of PHA at 5 μg/ml, 1 × 10^5^ PBMCs were cultured alone or in combination with hUCB-MSCs for 4 days, and then counted using CCK-8. *p < 0.05, ***p < 0.001 and ****p < 0.0001. The results showed are from more than three independent experiments. **(B,C)** 1 × 10^5^ CFSE-labeled CD4 or CD8 cells were stimulated with 10 μg/ml plate-bound CD3 antibodies and 1 μg/ml CD28 antibodies for 4 days. At the beginning of the stimulation period, add half number of hUCB-MSCs to the relevant wells. Flow cytometry was used to observe proliferation of CD4 and CD8 cells. **p < 0.01, ***p < 0.001 and ****p < 0.0001. The results showed are from more than three independent experiments.

The effect of hUCB-MSCs on the proliferation of PBMC was detected to investigate the immunoregulatory characteristics of hUCB-MSCs. It was found that hUCB-MSCs could inhibit the proliferation of PBMC stimulated by PHA when co-cultured under cell-cell contact for 4 days at different ratios (Figure 2A).

To investigate whether hUCB-MSCs can regulate T cell proliferation *in vitro*, we hatched CFSE-labeled CD4 and CD8 cells with hUCB-MSCs and measured 4 days later. Under the stimulation of CD3 and CD28 antibodies, we found that hUCB-MSCs were able to restrain the proliferation of CD4 and CD8 cells (Figures 2B,C). These data suggest that hUCB-MSCs can repress T cell proliferation *in vitro*.

### hUCB-MSCs Exhibit Higher Expression of Immunosuppressive Mediators in Response to IFN-γ

**FIGURE 3:**
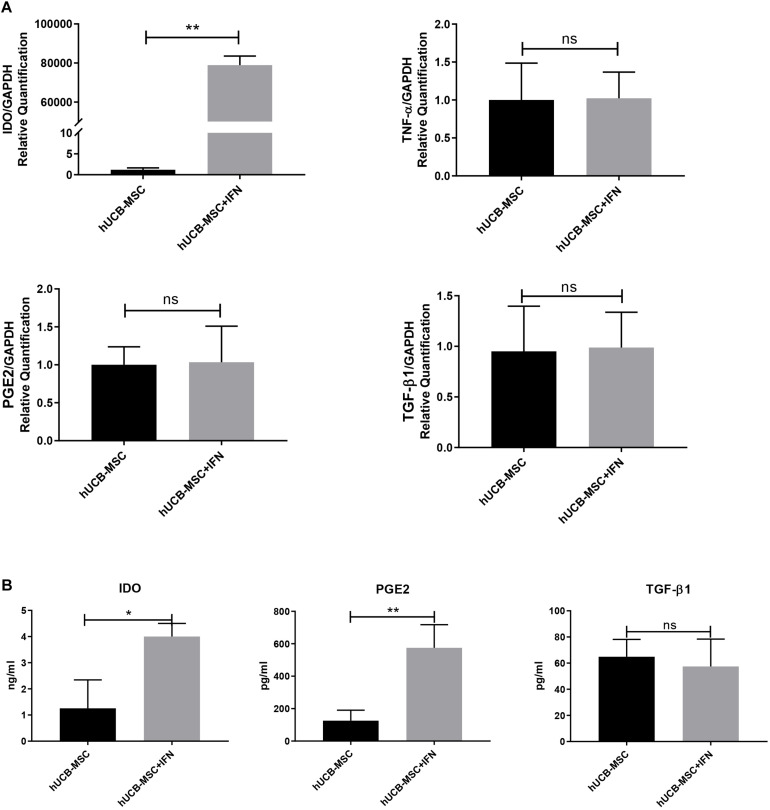
hUCB-MSCs show hyper-immunomodulatory potency with the stimulation of IFN-γ. **(A)** qPCR for IDO, TNF-α, PGE2, and TGF-β1 mRNA expression level in hUCB-MSCs with or without IFN-γ stimulation. **(B)** hUCB-MSCs were cultured in the presence of IFN-γ for 2 days, after which IDO, PGE2, and TGF-β1 levels in the supernatant were determined using ELISA. The results shown are from duplicate cultures performed in parallel. *p < 0.05, **p < 0.01, and ns, no significant difference.

The levels of TNF-α, interleukin (IL)-1β, IL-6, and especially IFN-γ were markedly increased after DSS induction (Gonzalez-Rey et al., 2009). The expression of secretory pro-inflammatory and immunosuppressive mediators was detected to observe the reaction of hUCB-MSCs to pro-inflammatory signals. We investigated changes in mRNA expression in hUCB-MSCs treated with IFN-γ for 2 days using qPCR. IDO was upregulated dramatically, whereas other pro-inflammation and immunosuppressive mediators such as TNF-α, PGE2, and TGF-β1 did not change significantly (Figure 3A). We measured the levels of the immunosuppressive mediators TGF-β1, IDO, and PGE2 in cell culture supernatant collected 2 days after IFN-γ treatment. Upon IFN-γ stimulation, hUCB-MSCs increased the secretion of IDO and PGE2, whereas IFN-γ stimulation did not induce the secretion of TGF-β1 in hUCB-MSCs (Figure 3B).

### hUCB-MSC Injection Protects Against DSS-Induced Colitis

After confirming the immunosuppressive activity of hUCB-MSCs *in vitro*, we inquiried whether there is a therapeutic effect of hUCB-MSCs on mouse model. Many chemical reagents can lead mice to develop colitis, the most commonly used one is DSS, which can simulate human IBD in mice, with regards to mucosal epithelial cell necrosis, loss of intestinal barrier function, and invasion of immune cells, such as macrophages and T cells (Wang et al., 2016).

**FIGURE 4:**
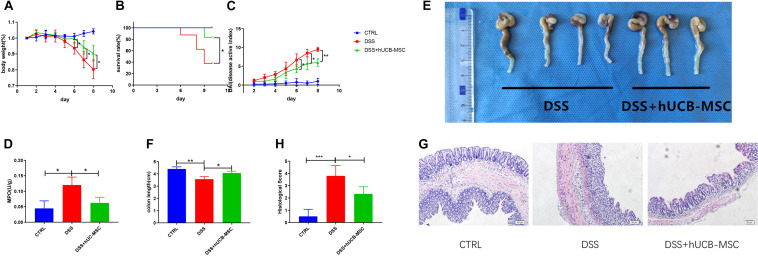
Treatment with hUCB-MSCs protects against DSS-induced acute colitis. Weight loss **(A)**, survival rate **(B)**, and disease activity index **(C)** were determined daily. MPO activity in colonic protein extracts were used to show neutrophil infiltration **(D)**. Colon length **(E,F)** and histopathological signs **(G,H)** were determined on day 10. The control group used tap water. *n* = 6–8 mice per group. *p < 0.05, **p < 0.01, ***p < 0.001. The magnification of HE staining images is 10 × 10.

A cute colitis was induced by 3% DSS in drinking water feeding C57BL/6 mice for 8 consecutive days. Figure 4A shows that hUCB-MSCs dramatically ameliorated the body weight reduction compared to the DSS group. We also found that injection of human hUCB-MSCs prevented mice from DSS-induced death (Figure 4B). hUCB-MSCs reduced the severity of DSS-induced colitis on the disease active index, as shown in Figure 4C. Moreover, hUCB-MSC-treated group exhibited a lower colonic MPO activity than DSS group, demonstrating less neutrophil invasion (Figure 4D). At day 10, all mice were euthanized and the colon length of each mouse was recorded. The degree of colon length shortening was relatively lesser in the hUCB-MSCs treated group than that in the group treated with DSS alone (Figures 4E,F). Analysis of distal colon sections from control group, DSS group, and DSS + hUCB-MSC group indicated that mice injected with hUCB-MSC exhibited much less destruction of the mucosal epithelium than the DSS group, such as reduced focal crypt lesions, goblet cell loss, and inflammatory cell infiltration, leading to a significant decrease in the histological score (Figures 4G,H).

**FIGURE 5:**
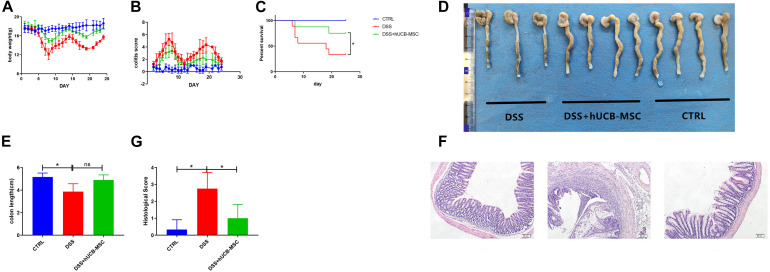
hUCB-MSC ameliorated DSS-induced chronic colitis. Body weight **(A)**, colitis score **(B)**, and survival rate **(C)** were determined daily. Colon length **(D,E)** and histopathological signs **(F,G)** were determined on day 25. *n* = 5–6 mice per group. *p < 0.05 and ns, no significant difference. The magnification of HE staining images is 10 × 10.

Chronic colitis was induced by 3% DSS in drinking water feeding C57BL/6 mice in two cycles. We observed mice showed continuous body weight reduction, diarrhea, and rectal bleeding (Figures 5A,B). Colon length shortening in DSS + hUCB-MSC group was less obvious than DSS group, but did not reach the significance threshold, probably because the sample size of the DSS group is too small (*n* = 3 on day 25) (Figures 5D,E). hUCB-MSC injections during the onset and the recurrent phase of colitis significantly reduced the clinical severity, representing body weight improvement, the frequency of diarrhea and hematochezia reduction, and survival rate increasing (Figures 5A–C). Moreover, hUCB-MSC infusions at the onset phase conferred protection to disease attack during the second round of DSS feeding to some extent (Figures 5A,B). hUCB-MSC injections during the second round of DSS administration almost abolished the clinical symptoms (Figures 5A,B). Histological examination of the colon represented that hUCB-MSC injection alleviated DSS-caused architectural dearrangements, epithelial necrosis, crypt abscesses, and lymphocytic infiltration (Figures 5F,G).

### hUCB-MSC Treatment Reduces T Cell Infiltration in Colon and Induces Tregs in Colitis

**FIGURE 6:**
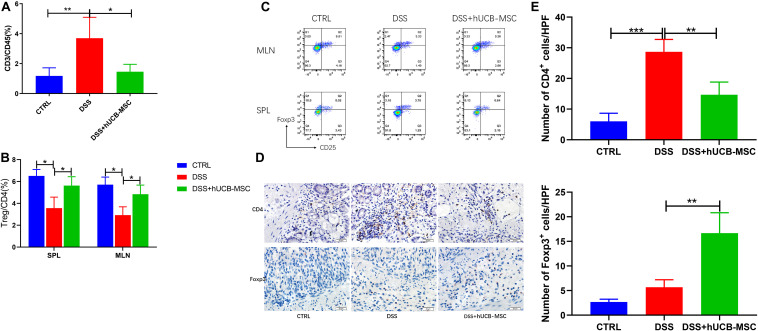
hUCB-MSC treatment reduced T cell infiltration in colon and induced Tregs in MLN, SPL, and colon. **(A)** Evaluation of T cell (%) infiltration to the colon. Flow cytometry analysis of CD3^+^ T cells gated in CD45^+^ population. **(B,C)** MLNs and splenocytes were analyzed for expression of Treg (staining for Foxp3 and CD25 in gated CD4^+^ T cells) via flow cytometry. *p < 0.05 and **p < 0.01. **(D)** Immunohistochemical staining (10 × 40) of CD4^+^ cells and Foxp3^+^ cells in colon tissues were showed. **(E)** Statistical results of immunohistochemical staining images for CD4^+^ cells and Foxp3^+^ cells. **p < 0.01 and ***p < 0.001.

In DSS induced colitis, the infiltration of T cells was analyzed by flow cytometry, as shown in Figure 6A. Very few T cells infiltrated the colon in the control group. When mice were treated with DSS, substantially more T cell infiltration occurred. Moreover, hUCB-MSC injections significantly alleviated T cell infiltration. Numbers of CD25^+^Foxp3^+^ Tregs in spleen and MLNs, especially the latter, which are major lymph nodes draining from the gut, of mice with colitis treated by hUCB-MSC were significantly increased, than untreated mice with colitis (Figures 6B,C). To observe whether hUCB-MSCs decreased T cell infiltration in the colon and stimulated Treg activation, we measured the number of T cells and Foxp3^+^ cells after injection of hUCB-MSCs via immunohistochemical staining. The control group showed sparse T cells and Foxp3^+^ cells (Figures 6D,E). There were many T cells in the colon of the DSS group, but still only few Foxp3^+^ cells. The number of T cells present in the colon of the hUCB-MSC-injected group was less than those in the DSS group, in contrast, more Foxp3^+^ cells were observed. Tregs can be activated by hUCB-MSCs *in vivo*, and Tregs could be used as a therapeutic target.

## Discussion

The present study revealed the efficacy of hUCB-MSCs on a mouse model of IBD induced by DSS. The restoration of immune tolerance by re-establishment of the Treg repertory may be an attractive mechanism by which MSCs exert their protective effect (Gonzalez-Rey et al., 2009).

In recent years, a major breakthrough has been the apparent immunosuppressive and anti-inflammatory functions of MSCs both *in vitro* and *in vivo*. Mechanisms by which MSCs alleviate inflammation and immune response have been well understood (Wang et al., 2016). In this study, we showed that hUCB-MSCs strongly suppressed PBMC proliferation and inhibited CD4^+^ and CD8^+^ T cell proliferation when they were cultured together with PBMCs.

In this study, we also demonstrated that stimulation with IFN-γ elevated the mRNA expression level of IDO dramatically and increased the concentration of IDO and PGE2, which are two important mediators by which MSCs inhibit T cells, in the cell culture supernatant from hUCB-MSCs. In particular, IDO plays an important role in immunoregulation mediated by human MSCs. IDO secreted by human MSCs was essential for suppressing the expansion of Th1 cells stimulated by IFN-γ (Qiu et al., 2017). According to previous studies, PGE2 mediate major immunodepression functions of adipose-derived MSCs and BMSCs, including proliferation of T cells and exerting functions of dendritic cells (Yañez et al., 2010). PGE2 secreted by MSC can transform inflammatory microenvironment into anti-inflammatory, changing the type of cytokines secreted by T cells and dendritic cells (Qiu et al., 2017). In addition, Chen et al. (2010) has reported that PGE2 plays an irreplaceable role in the immunodepression effect of hUCB-MSCs, because once secretion of PGE2 was restrained, immunodepression functions of hUCB-MSCs almost completely disappeared. What’s more, PGE2 works with IDO synergistically. Therefore, irritation with IFN-γ enhanced the immunosuppressive effect of hUCB-MSCs. These findings suggest that IFN-γ, as a key feedback signal molecule in the interaction between immune cells and MSCs, has significance in immunodepression mediated by MSCs. In DSS-induced colitis, IFN-γ concentrations in both the serum and colon increased (Gonzalez-Rey et al., 2009), further enhancing the immunosuppressive effect of injected hUCB-MSCs.

The objective of this research was to confirm if administration of hUCB-MSCs relieved DSS-induced acute and chronic mouse colitis. Human MSCs are exempted of immune rejection because of low expression of the major histocompatibility complex II (Chamberlain et al., 2007). Therefore, we performed experiments using wild type mice to examine whether hUCB-MSCs had anti-inflammatory functions *in vivo*. We found that intraperitoneal injection of hUCB-MSCs in mice with DSS-induced colitis reduced the high mortality caused by this syndrome and alleviated the disease symptoms i.e., weight loss, bleeding, and diarrhea in comparison with the DSS group. In IBD, immune cells infiltration to lamina propria causes mucosal inflammation and thus, a decrease in the colon length in terms of macroscopic changes after colitis is noted (Turner, 2009). Our findings revealed that in DSS group, colon length was shorter compared to that of the hUCB-MSC-treated group. In this research, histopathological examination revealed that in hUCB-MSCs treated group, immune cell infiltration to the intestinal mucosa was reduced apparently, which proved a relief of immune responses attributed to hUCB-MSC infusion. On the contrary, mucosal decomposition in DSS group were extremely obvious as the crypt structure was damaged and inflammatory cell infiltration could be seen extensively.

Multiple regulatory mechanisms collaborate on the maintenance of intestinal homeostasis, and once disrupting certain pathway may cause abnormal immune responses to intestinal environments, thus, leading to occurrence of IBD. Colonic Tregs identify food and commensal flora and inhibit immune activities specific to them (Himmel et al., 2012; Tanoue et al., 2016). A previous study has revealed that intestinal homeostasis connected with Treg activation closely (Maynard et al., 2007). In particular, CD25^+^Foxp3^+^ Tregs are capable of suppressing activation, expansion and taking effects of a variety of immune cells, containing antigen-presenting cells, natural killer cells, and T, B lymphocytes, so they are powerful mediators of main immune tolerance of periphery (Sakaguchi et al., 2010). A subtle balance between T effectors and Tregs has been constructed in intestinal homeostasis of health control, however, in patients with IBD, that balance has been struck, the number of Tregs decreases and the number of T effectors increases (Ueno et al., 2018). According to flow cytometry results and immunohistochemistry, hUCB-MSCs prevented infiltration of CD4^+^ T cells and enhanced production of Foxp3^+^ Tregs in the colon. The percentage of CD25^+^Foxp3^+^ Tregs showed an increase in the MLNs and spleen of the DSS + hUCB-MSC group than that of the DSS group. Above findings confirmed the induction of Tregs by hUCB-MSCs may also be the mechanism of hUCB-MSCs to perform immunosuppressive functions in DSS-induced colitis.

We have demonstrated that hUCB-MSCs are able to inhibit PBMC proliferation *in vitro*, notably T cells. When stimulated by IFN-γ, hUCB-MSCs exert stronger immunosuppressive effects by secreting more PGE2 and IDO. These two soluble factors play important roles in Treg and T effector cell balance, which regulates various aspects of the immune response (Le Blanc and Mougiakakos, 2012; Melief et al., 2013; Heidari et al., 2018). It has been reported that PGE2 can facilitate the production of Tregs (Qiu et al., 2017). Kim et al. (2013) revealed that nucleotide-binding oligomerization domain-containing protein two activation leads to hUCB-MSCs secreting PGE2, which results in an elevation of the Treg population. Zhang Q. et al. (2018) suspected that IDO might play an important part in the process of transformation of PBMC into Tregs. Kadle et al. (2018) described a significant role of IDO in rat MSC immunosuppression. They discovered that suppressing IDO substantially decreased the expansion of Tregs, and the suppressive functions of MSCs in coculture disappeared basically. This proves that IDO plays an essential part in MSC-mediated immunomodulation, which is achieved by Tregs at least in part. Further differentiation of Tregs facilitates MSCs to generate immune tolerance, thus reinforcing the suppressive effect of MSCs in immune activities (Madrigal et al., 2014; Cao et al., 2015; Lee et al., 2015).

Human umbilical cord blood derived-mesenchymal stem cells relieved DSS-induced colitis in mice by elevating the number of Treg population, thereby providing an attractive therapeutic strategy. In conclusion, our results suggest that hUCB-MSCs may be promising alternatives for cell-based treatment in patients with IBD.

## Data Availability Statement

The original contributions presented in the study are included in the article, further inquiries can be directed to the corresponding authors.

## Ethics Statement

The animal study was reviewed and approved by the Animal Ethics Committee of the First Hospital of Jilin University.

## Author Contributions

KM and LZ participated in the experiments and analyzed the data. KM wrote the manuscript. YL did literature review. HX and NZ conceived and designed this study. The corresponding authors were responsible for submitting all materials. All authors reviewed and approved the final manuscript.

## Conflict of Interest

The authors declare that the research was conducted in the absence of any commercial or financial relationships that could be construed as a potential conflict of interest.
